# Distinguishing Genetic Alterations Versus (Epi)Mutations in Silver–Russell Syndrome and Focus on the *IGF1R* Gene

**DOI:** 10.1210/clinem/dgae730

**Published:** 2024-10-16

**Authors:** Alessandro Vimercati, Pierpaola Tannorella, Sara Guzzetti, Luciano Calzari, Davide Gentilini, Emanuela Manfredini, Giulia Gori, Rossella Gaudino, Vincenzo Antona, Maria Piccione, Cecilia Daolio, Renata Auricchio, Fabio Sirchia, Antonella Minelli, Elena Rossi, Melissa Bellini, Giacomo Biasucci, Annalisa Russo Raucci, Gabriella Pozzobon, Giuseppa Patti, Flavia Napoli, Lidia Larizza, Mohamad Maghnie, Silvia Russo

**Affiliations:** Research Laboratory of Medical Cytogenetics and Molecular Genetics, IRCCS Istituto Auxologico Italiano, 20145 Milan, Italy; Research Laboratory of Medical Cytogenetics and Molecular Genetics, IRCCS Istituto Auxologico Italiano, 20145 Milan, Italy; Research Laboratory of Medical Cytogenetics and Molecular Genetics, IRCCS Istituto Auxologico Italiano, 20145 Milan, Italy; Bioinformatics and Statistical Genomic Unit, IRCCS Istituto Auxologico Italiano, 20145 Milan, Italy; Bioinformatics and Statistical Genomic Unit, IRCCS Istituto Auxologico Italiano, 20145 Milan, Italy; Department of Brain and Behavioral Sciences, University of Pavia, 27100 Pavia, Italy; Research Laboratory of Medical Cytogenetics and Molecular Genetics, IRCCS Istituto Auxologico Italiano, 20145 Milan, Italy; Medical Genetics Unit, Meyer Children's Hospital IRCCS, 50139 Florence, Italy; Pediatric Unit, Department of Surgical Sciences, Dentistry, Gynecology and Pediatrics, University of Verona, 37129 Verona, Italy; Department of Health Promotion, Mother and Child Care, Internal Medicine and Medical Specialties “G. D'Alessandro,” University of Palermo, 90127 Palermo, Italy; Medical Genetics Unit Department of Health Promotion, Mother and Child Care, Internal Medicine and Medical Specialties, University of Palermo, 90127 Palermo, Italy; Department of Pediatrics, Fondazione IRCCS San Gerardo dei Tintori, 20900 Monza, Italy; European Laboratory for the Investigation of Food Induced Diseases, Department of Translational Medical Science, Section of Pediatrics, University Federico II, 80131 Naples, Italy; Medical Genetic Unit, Department of Diagnostic Medicine, IRCCS San Matteo Hospital Foundation, 27100 Pavia, Italy; Department of Molecular Medicine, University of Pavia, 27100 Pavia, Italy; Medical Genetic Unit, Department of Diagnostic Medicine, IRCCS San Matteo Hospital Foundation, 27100 Pavia, Italy; Department of Molecular Medicine, University of Pavia, 27100 Pavia, Italy; IRCCS Mondino Foundation, 27100 Pavia, Italy; Pediatrics and Neonatology Unit, Gugliemo da Saliceto Hospital, 29121 Piacenza, Italy; Pediatrics and Neonatology Unit, Gugliemo da Saliceto Hospital, 29121 Piacenza, Italy; Department of Medicine and Surgery, University of Parma, 43125 Parma, Italy; Division of Genetics and Cell Biology and Laboratory of Clinical Molecular Biology and Cytogenetics, Unit of Genomics for Human Disease Diagnosis, IRCCS San Raffaele Scientific Institute, 20132 Milan, Italy; Pediatric Endocrinology Unit, San Raffaele Hospital, 20132 Milan, Italy; Paediatric Endocrinology Unit, IRCCS Istituto Giannina Gaslini, 16147 Genoa, Italy; Department of Neuroscience, Rehabilitation, Ophthalmology, Genetics, Maternal and Child Health, University of Genoa, 16147 Genoa, Italy; Paediatric Endocrinology Unit, IRCCS Istituto Giannina Gaslini, 16147 Genoa, Italy; Research Laboratory of Medical Cytogenetics and Molecular Genetics, IRCCS Istituto Auxologico Italiano, 20145 Milan, Italy; Paediatric Endocrinology Unit, IRCCS Istituto Giannina Gaslini, 16147 Genoa, Italy; Department of Neuroscience, Rehabilitation, Ophthalmology, Genetics, Maternal and Child Health, University of Genoa, 16147 Genoa, Italy; Research Laboratory of Medical Cytogenetics and Molecular Genetics, IRCCS Istituto Auxologico Italiano, 20145 Milan, Italy

**Keywords:** Silver–Russell syndrome, *IGF1R*, *IGF2*-PLAG1-HMGA2 axis, familial cases, body asymmetry, (epi)genetic phenotype

## Abstract

**Context:**

Silver–Russell Syndrome (SRS) is a growth retardation disorder characterized by pre- and postnatal growth failure, relative macrocephaly at birth, prominent forehead, body asymmetry, and feeding difficulties. The main molecular mechanisms are imprinting alterations at multiple loci, though a small number of pathogenic variants have been reported in the SRS genes *IGF2*-*PLAG1*-*HMGA2* and *CDKN1C*. However, around 40% of clinically suspected SRS cases do not achieve a molecular diagnosis, highlighting the necessity to uncover the underlying mechanism in unsolved cases.

**Objective:**

Evaluate the frequency of genetic variants in undiagnosed SRS patients [Netchine–Harbison Clinical Scoring System (NH-CSS) ≥ 4], and investigate whether (epi)genetic patients may be distinguished from genetic patients.

**Methods:**

One hundred thirty-two clinically SRS patients without (epi)genetic deregulations were investigated by whole-exome (n = 15) and targeted (n = 117) Sequencing. Clinical data from our cohort and from an extensive revision of the literature were compared.

**Results:**

Pathogenic variants were identified in 9.1% of this cohort: 3% in *IGF2*, *PLAG1*, and *HMGA2* genes and 3% in the *IGF1R* gene, associated with IGF-1 resistance (IGF1RES), an SRS differential diagnosis. Overall, *IGF2*-*PLAG1*-*HMGA2* and *IGF1R* account for 3.6% of SRS with NH-CSS score ≥ 4. A clinical cross-comparison of (epi)genetic vs genetic SRS underlined (epi)genotype-phenotype correlation highlighted the prevalence of body asymmetry and relative macrocephaly in mosaic (epi)genetic SRS and recurrence of genetic familial cases. Furthermore, overlapping features were evidenced in (epi)genetic SRS and IGF1RES patients.

**Conclusion:**

Our study explores the frequency of genetic SRS, underscores body asymmetry as a distinctive phenotype in (epi)genetic SRS and suggests *IGF1R* sequencing in a SRS diagnostic flowchart.

Silver–Russell syndrome (SRS) is a rare (1:30.000-100.000) imprinting disorder characterized by severe prenatal and postnatal growth retardation (PNGR), relative macrocephaly at birth associated with a triangular face and a prominent forehead, body asymmetry, and feeding difficulties. Clinical diagnosis is based on the occurrence of at least 4 out of 6 clinical signs, in accordance with the Netchine–Harbison Clinical Scoring System (NH-CSS), but molecular testing is recommended in patients with ≥3/6 criteria (1). The etiology of SRS mainly consists in the deregulation of imprinting at specific loci: 30% to 60% of patients, defined as SRS type 1 (MIM#180860), have loss of methylation of the paternal allele at *H19/IGF2:IG-DMR* in the 11p15.5 chromosomal region (IC1_LoM), while 5% to 10% (SRS type 2, MIM#618905) have maternal uniparental disomy of chromosome 7 (UPD(7)mat, involving the *GRB10:alt-TSS-DMR*, *PEG10:TSS-DMR*, and *MEST:alt-TSS-DMR*) (1-3). Furthermore, a small number of cases with an SRS-like presentation display epimutations or UPD(14)mat at the *MEG3:TSS-DMR* (14q32) associated with Temple syndrome (MIM#616222) (4, 5) or UPD(20)mat associated with Mulchandani–Bhoj–Conlin syndrome (MIM#617352) (6, 7). Rare genetic causes are also reported: pathogenic variants affecting the genes of the *IGF2*-*PLAG1-HMGA2* pathway have been associated with a diagnosis of SRS type 3 (MIM#616489), SRS type 4 (MIM#618907), and SRS type 5 (MIM#618908), respectively. This pathway plays a crucial role in the regulation of physiological fetal and postnatal growth, and disruption of each involved gene affects the expression of *IGF2* as LoM at *H19/IGF2:IG-DMR* (8). In addition, very rare pathogenic variants within the PCNA-binding domain of *CDKN1C* are responsible for a severe differential diagnosis of SRS, named IMAGE syndrome (MIM#614732). The limited number of cases so far described has not enabled a complete definition of the phenotype of these genetic SRS subtypes (9). Overall, in about 40% of patients with a clinical suspicion of SRS, the molecular defect remains to be ascertained (1, 2, 6-8). With the implementation of next-generation sequencing (NGS) technology, various reports have been published (10-12), bringing to light a broad spectrum of monogenic diseases that exhibit clinical features overlapping with SRS. IGF1RES (MIM#612626), SHORT syndrome (MIM#269880), 3-M syndrome (MIM#273750), and Mulibrey nanism (MIM#253250), whose clinical presentation is sometimes hard to distinguish from SRS (1, 13, 14), are those reported at a higher frequency.

Here we refer to a cohort of 132 SRS patients with NH-CSS ≥ 4 but without a molecular diagnosis. All were investigated for pathogenic variants in the main SRS genes, and a small subset by whole-exome sequencing (WES) and single nucleotide polymorphism (SNP) array. The application of this flowchart allowed us to assign a diagnosis to 9.1% of cases and to highlight novel genotype-phenotype correlations.

## Materials and Methods

### Study Cohort

A cohort of 324 patients, scored as NH-CSS ≥ 3, were referred to our center for SRS genetic testing from 2006 to 2023. Application of our reported diagnostic flowchart (6) led to the detection of 73/324 IC1_LoM (22.5%), 21/324 UPD(7)mat (6.5%), 7/324 Temple syndrome (2.1%), 3/324 UPD(20)mat (0.9%) by mass spectrometry-multiplex ligation-dependent probe amplification (MLPA) (MRC Holland, Amsterdam, Netherlands). Furthermore, 3 chromosomal rearrangements at the 11p15.5 region (0.9%) and a *NSD1* duplication were identified. Out of 324 patients, 221 had an NH-CSS score ≥ 4. Among these, 61 had IC1_LoM, (27.6%), 18 UPD(7)mat (8.1%), 4 Temple syndrome (1.8%), 3 UPD(20)mat (1.3%), and 3 11p15.5 rearrangements (1.3%). In sum, in our global SRS cohort imprinting is deregulated in about 33% of cases, rising to 40% when only patients with NH-CSS score ≥ 4 are considered. Overall, 132 patients with an NH-CSS score ≥ 4 and without a genetic diagnosis were enrolled in this study. Chromosomal abnormalities were excluded using karyotyping and comparative genomic hybridization (CGH) array 60 K, while *CDKN1C* variants were ruled out by Sanger sequencing. Clinical information was collected from patients’ attending physicians, and written informed consent to the genetic test was received from all patients or parents. The patients’ parents consented to have their children's image published. The Ethical Committee of IRCSS Istituto Auxologico Italiano approved the study (CE: 2017_05_16_05).

### MLPA

*IGF1R* and *HMGA2* copy number variations were assessed by MLPA using the P217 IGF1R and the P323 CDK4-HMGA2-MDM2 probemix. The analyses were performed according to manufacturers’ protocols. In each experiment 4 control samples were included. Raw data were analyzed using Coffalyser.Net software (version 140,701, MRC Holland).

### NGS Analysis

In accordance with the manufacturer's protocols, DNA was extracted from peripheral blood lymphocytes (Wizard Genomic DNA Purification Kit, Promega). NGS analysis was conducted using 2 approaches: (1) WES with the SureSelect Human All Exon V7 library (Agilent) and (2) sequencing of a small gene panel comprising 3 SRS-associated genes (*IGF2*, *PLAG1*, and *HMGA2)* and *IGF1R*. WES bioinformatic analyses were performed according to a previously published pipeline (15). Libraries for amplicon-based sequencing were generated using the Nextera XT DNA Library Prep Kit (Illumina, San Diego, CA) and sequenced with an Illumina Miseq sequencer. Bioinformatic analyses were conducted using the default parameters of Illumina's Miseq Reporter software (v.2.6.2): demultiplexed reads were aligned to the reference genome (hg19) using the Burrows-Wheeler Aligner, and variant calls were identified using the Genome Analysis ToolKit (v1.6) Unified Genotyper. Variant annotation was performed using the wANNOVAR tool (16). To disclose causative variants, a virtual panel of 2508 growth-related genes was designed by reviewing the literature and using PanelApp (17). All variants identified by these 2 approaches were filtered by minor allele frequency < 1% in the 1000 Genomes, Genome Aggregation Databases, and Exome Aggregation Consortium databases. In silico prediction of missense variants’ pathogenicity was performed by combining the PolyPhen-2, SIFT, and CADD algorithms. The interpretation of the variants was based on the classification by the InterVar, VarSome, and Franklin by Genoox databases (18, 19) in accordance with the American College of Medical Genetics and Genomics/Association for Molecular Pathology guidelines (20, 21). All the variants reported here were confirmed by Sanger sequencing.

### CGH array and SNP array

Whole-genome array-CGH analysis was performed using the 180 K platform (kit 4 × 180 K CGH + SNP, AGILENT), with an average resolution of 40 kb in optimal conditions, to detect copy number variants (CNVs) and loss of heterozygosity. Labeling and hybridization were performed according to the manufacturer's protocol and CNVs were detected by the Agilent Cytogenomics 5.0.2.5 analysis software. The map positions refer to the Human Genome Building 37 (hg19) assembly.

Infinium HD Assay Ultra with Illumina Infinium CytoSNP-850 K v1.4 BeadChips was performed to detect CNVs (duplications, deletions, loss of heterozygosity) in accordance with the manufacturer's instructions. The data were imported from iScan Control Software into GenomeStudio 2.0 Genotyping Module Software provided by Illumina for analysis.

In both cases, the map positions refer to the Human Genome Building 37 (hg19) assembly, and a CNV was identified by at least 3 consecutive experiments with locus-specific probes. Detected CNVs were compared with the Database of Genomic Variants (http://projects.tcag.ca/variation/, release March 2016) to exclude common copy number polymorphisms (minor allele frequency >1%). The establishment of CNV pathogenicity was made following the American College of Medical Genetics recommendations (22)

### Statistical Analysis

Fisher's exact test was used to assess differences in the frequency of clinical features between (epi)genetic- and genetic-based SRS and between (epi)genetic SRS and *IGF1R* patients. Statistical analysis was performed using the Graph Pad Prism 7 program. A *P*-value ≤ .05 was considered statistically significant.

## Results

### WES and SNP Array Molecular Analyses

WES trio was performed on 15 out of 132 patients. Patients were selected on the basis of their clinical features and availability. Overall, 6 out of 15 unrelated SRS patients achieved a diagnosis after WES, including 1 inherited variant in the *PLAG1* gene and 2 variants in the *IGF1R* gene (1 de novo and 1 inherited); 1 de novo variant in the *FGFR3* gene; and 2 children with autosomal recessive inheritance in the *CCDC8* and *SBDS* genes. Table 1 reports the identified variants, classified according to the American College of Medical Genetics and Genomics criteria, the mode of inheritance of the associated disease, and the results of the segregation analysis. Due to a discrepancy between patient's phenotype and candidate gene's phenotype, 2 cases remain with uncertain diagnoses: SRS91 with a compound heterozygous genotype of a pathogenic and an unknown significance (VUS) variant in *BRAT1* gene associated with NEDCAS syndrome (MIM#618056) (23) and SRS08, carrier of a paternally inherited VUS variant in the *CHD7* gene associated with CHARGE syndrome (MIM#214800) (24, 25).

**Table 1.** Overview of the variants detected by WES and using an NGS amplicon-based approach for the SRS genes (reference genome hg19)

Gene	Patient	Method	Coding DNA level	Protein level	Mendelian trait	Inheritance	gnomAD Exomes frequency	CADD	Polyphen	SIFT	ACMG classification
*IGF2*	SRS05	NGS Amplicon	NM_000612.6: c.-6-2A > T	p.?	AD, het	Pat*^a^*	—	33	—	—	Pathogenic (PVS1, PM2, PP1, PP4)
*HMGA2*	SRS75	NGS Amplicon	NM_003483.6: c.41C > G	p.(Ser14Ter)	AD, het	de novo	—	36	—	—	Pathogenic (PVS1, PS2, PM2, PP4)
*PLAG1*	SRS44	WES	NM_002655: c.671G > A	p.(Arg224Gln)	AD, het.	mat*^a^*	—	26.2	1 (D)	0 (D)	Likely pathogenic (PM1, PM2, PP1, PP3, PP4, BP1)
*PLAG1*	SRS90	NGS Amplicon	NM_002655: c.610_612del	p.(Met204del)	AD, het	mat*^a^*	—	—	—	—	Likely pathogenic (PM1, PM2, PM4, PP1, PP4)
*IGF1R*	SRS67	WES	NM_000875: c.1079T > C	p.(Leu360Ser)	AD, het.	mat*^a^*	—	29.5	0.97 (D)	0 (D)	Likely pathogenic (PM1, PM2, PP1, PP2, PP3, PP4)
*IGF1R*	SRS114	WES	NM_000875: c.1363T > C	p.(Cys455Arg)	AD, het.	de novo	—	26.3	1 (D)	0 (D)	Pathogenic (PS2, PM1, PM2, PP2, PP3, PP4)
*CCDC8*	SRS74	WES	NM_032040: c.451dupG	p.(Glu151GlyfsTer30)	AR, hom.	mat/pat*^c^*	—	—	—	—	Pathogenic (PVS1, PM2, PM3, PP4)
*FGFR3*	SRS03	WES	NM_000142.5: c.1663G > T	p.(Val555Leu)	AD, het.	de novo	—	25.9	1 (D)	0.001 (D)	Likely pathogenic (PS2, PM2, PP3)
*SBDS*	SRS104	WES	NM_016038: c.258 + 2T > C c.128 + 6T > C	p.? p.?	AR, comp. het	pat mat	0.00388 0.00000797	33	—	—	Pathogenic (PVS1, PS3)
								23.2	—	—	Likely pathogenic (PM2, PM3, PP3)
*BRAT1*	SRS91	WES	NM_152743: c.638dupA c.1892C > T	p.(Val214GlyfsTer188) p.(Thr631Met)	AR, comp. het.	mat pat	0.000264 0.0000436	—	—	—	Pathogenic (PVS1, PM2)
								12.7	0.865 (D)	0.033 (D)	Uncertain significance (PM2, PM3, BP1, BP4)
*CHD7*	SRS08	WES	NM_017780: c.5927G > C	p.(Arg1976Pro)	AD, het	pat*^b^*	—	34	1 (D)	0 (D)	Uncertain significance (PM2, PP3)

Abbreviations: ACMG, American College of Medical Genetics and Genomics; AD, autosomal dominant; AR, autosomal recessive; comp. het., compound heterozygous; D, damaging; gnomAD, Genome Aggregation Databases; het, heterozygous; mat, maternal; NGS, next-generation sequencing; pat, paternal; SRS, Silver–Russell syndrome; T, tolerated; WES, whole-exome sequencing.

^*a*^Affected parents.

^*b*^Carrier.

^*c*^Consanguineous parents.

These 2 patients and the 7 undiagnosed WES-enrolled patients were then investigated using a high-resolution SNP or CGH array to comprehensively complete the mutational screen. Case SRS84 was found to harbor a de novo deletion of 206 Kb at 19q, arr[GRCh37] 19q13.33(48192995_48399399)x1 dn, which involves the entire *CRX* gene associated with cone-rod retinal dystrophy-2 (MIM#120970) and partially the *BICRA* gene (from exon 8 to 15) associated with Coffin-Siris syndrome 12 (CSS12, MIM#619325), both autosomal dominant pathologies (Supplementary Fig. S1) (26). No chromosomal rearrangements were revealed in the other cases.

**Figure 1.**
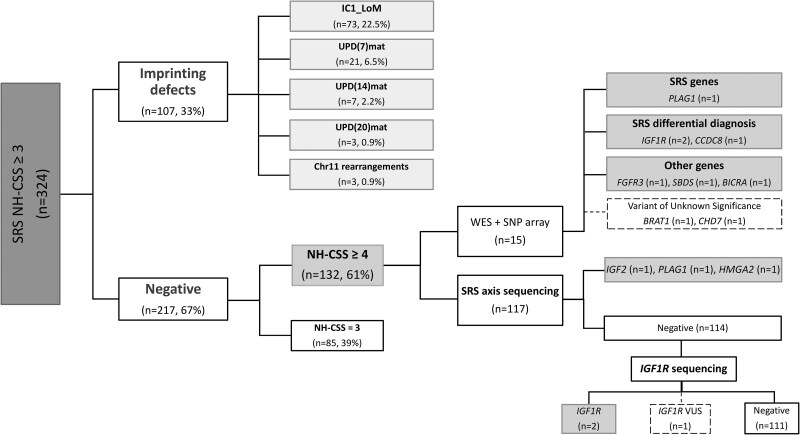
Flowchart of the molecular study. From 2006 to 2023, a total of 324 patients with suspected SSRS and a NH-CSS score of ≥ 3 were referred to our laboratory for genetic testing. All patients underwent methylation analysis for the 11p15.5 region and chromosomes 7, 14, and 20, revealing imprinting deregulation in 107 patients. Among the remaining 217 patients without a diagnosis, 132 patients with NH-CSS ≥ 4 were included in this study. Whole-exome sequencing and single nucleotide polymorphism array analysis were performed in 15 of these cases, uncovering causative molecular defects in 7 of them and identifying VUCs in 2 additional patients. The remaining 117 patients underwent sequencing of SRS axis-related genes, which resulted in a diagnosis for 3 cases. Subsequently, in 114 undiagnosed patients, sequencing of the *IGF1R* gene identified 2 causative variants and 1 VUS. Abbreviations: NH-CSS, Netchine–Harbison Clinical Scoring System; SRS, Silver–Russell syndrome; VUS, variant of uncertain significance.

The flowchart in Fig. 1 illustrates the molecular workup of patients and the achieved diagnosis.

### *IGF2-PLAG1-HMGA2* Pathway: Identification of New Genetic Defects

In the remaining 117 patients, sequencing of the *IGF2*, *PLAG1*, and *HMGA2* genes by an amplicon-based approach (Fig. 1) revealed 2 pathogenic variants and 1 likely pathogenic variant (Table 1). Specifically, SRS05 was a carrier of an *IGF2* splicing variant inherited from her affected father and predicted to disrupt the acceptor site upstream exon 2 of the gene; SRS75 harbored a de novo nonsense variant in the *HMGA2* gene; and SRS90 inherited from his affected mother a *PLAG1* in-frame deletion variant.

### Clinical Evaluation

**Figure 2.**
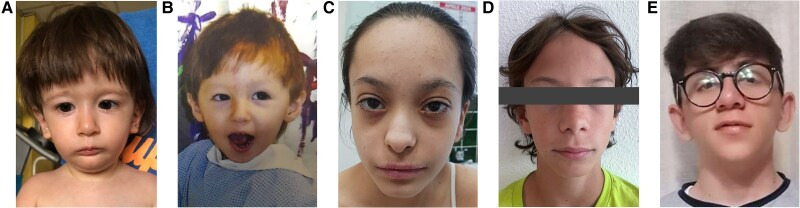
Photographs of patients (A) SRS05 with *IGF2* variant; (B) SRS44 with *PLAG1* missense variant at the age of 2 years; SRS67 (C) and SRS114 (D) with *IGF1R* variant and (E) SRS08 with a *CHD7* variant of unknown significance.

Table 2 sums up the clinical features of all SRS patients with an identified molecular alteration, including the affected parents (*IGF1R* cases are discussed in detail later). SRS facial features of a few patients are displayed in Fig. 2. Concerning SRS90's mother with a *PLAG1* variant (not indicated in the table), it is only known that she experienced growth difficulties in infancy with a final height of 147 cm [−2.51 SD score (SDS)] and exhibited a typical facies, characterized by a triangular face and a protruding forehead. Auxological parameters at birth and at last evaluation are reported for each patient: only the girl with a *FGFR3* variant was not born small for gestational age (SGA), and 8 out of 10 exhibited relative macrocephaly at birth, while body asymmetry has been described only in 1 patient. Furthermore, SRS facies, digital anomalies, and hypotonia were observed in the majority of the cases.

**Table 2.** Clinical features of patients identified by NGS analysis, excluding *IGF1R* patients

Patient	SRS05	SRS05 father	SRS44	SRS44 mother	SRS90	SRS75	SRS74
Gene	*IGF2*	*IGF2*	*PLAG1*	*PLAG1*	*PLAG1*	*HMGA2*	*CCDC8*
Genetic diagnosis	Silver–Russell type 3 (#616489)	Silver–Russell type 3 (#616489)	Silver–Russell type 4 (#618907)	Silver–Russell type 4 (#618907)	Silver–Russell type 4 (#618907)	Silver–Russell type 5 (#618908)	3 M syndrome 3 (#614145)
Sex	Female	Male	Male	Female	Male	Male	Male
IUGR	X	NA	X	X	X	X	X
Gestational age	36	preterm	39	39	37	38 + 6	41
BW in g (SDS)	1200 (−3.3)	800	1990 (−3.44)	2200 (−2.44)	1750 (−2.85)	2110 (−2.74)	2730 (−2.3)
BL in cm (SDS)	37 (−3.81)	NA	43 (−3.61)	NA	45 (−1.73)	45 (−2.38)	46 (−3.1)
BOFC in cm (SDS)	33 (0.3)	NA	32 (−2.3)	NA	34.5 (0.6)	33 (−1.11)	35 (−0.18)
Age at last evaluation	1y	36y	8y	42y	4y	7.5y	8m
Height in cm (SDS)	65.5 (−2.9)	152 (−3.4)	115 (−2.34)	148.7 (−2.25)	92.5 (−2.38)	111 (−2.56)	60 (−4.83)
Weight in kg (SDS)	5.8 (−4.98)	38.5 (−5.03)	15.2 (−4.93)	39 (−3.31)	10 (−5.15)	18 (−2.48)	7.5 (−1.69)
OFC in cm (SDS)	41 (−3.42)	50 (−3.43)	48 (−3.11)	NA	46.5 (−2.59)	51 (−0.85)	44.3 (−0.59)
B-Relative Macrocephaly	X	NA	—	NA	X	X	X
Feeding difficulties	X	X	X	X	X	—	X
Protruding forehead	X	X*^b^*	X	X	—	X	X
Body asymmetry	—	—	—	—	—	—	—
NH-CSS	5/6	4/5	4/6	4/5	4/6	4/6	5/6
Triangular face	X	X	X	X	X	X	X
Micrognathia	X	X	—	X	X	X	X
Thin lips	X	X	X	—	—	X	X
Down-turned mouth	X	—	X	X	X	—	—
Ogival palate	X	—	X	X	—	—	—
Other dysmorphisms	Short palpebral fissures	DsPF	Crowded teeth	Teeth anomalies	Trigonocephaly	Short palpebral fissures, short philtrum	Plagiocephaly
Digital anomalies	—	—	Cli + Syn	—	Cli	Cli + Bra	—
Small hand/feet	X	X	—	—	—	X	X
Ligamentous laxity	X	—	—	—	—	—	X
Hypotonia	—	—	X	X	—	—	X
Psychomotor delay	MD + SD	Mild ID	MD + SD	—	—	—	MD
Other features	—	Learning difficulties	GER, hypospadias, ADHD, normal GH and IGF-1 levels, DBA	—	—	Prominent heels, autism, normal GH and IGF-1 levels, DBA	GER, HypoE, multiple Mongolian spots, normal GH levels

Patient	SRS03	SRS104	SRS91	SRS08	SRS84
Gene	*FGFR3*	*SBDS*	*BRAT1*	*CHD7*	19q13.33 deletion
Genetic diagnosis	Hypochondroplasia (#146000)	Shwachman–Diamond syndrome 1 (#260400)	NEDCAS (#618056)	CHARGE syndrome, atypical (#214800)	CSS12 (#619325), cone-rod retinal dystrophy (#2120970)
Sex	Female	Male	Male	Male	Female
IUGR	—	X	X	—	X
Gestational age	39 + 4	33 + 2	37 + 5	38 + 6	32
BW in g (SDS)	3190 (−0.39)	1210 (−2.23)	2065 (−2.32)	2200 (−2.54)	800 (−2.79)
BL in cm (SDS)	48 (−1.04)	36 (−3.09)	37 (−4.55)	45 (−2.3)	30 (−4.44)
BOFC in cm (SDS)	36 (1.59)	29 (−1.33)	31 (−2.2)	32.7 (−1.35)	25.5 (−2.65)
Age at last evaluation	9y	3.5y	11y	10.5y	10y
Height in cm (SDS)	118 (−2.56)	82 (−3.9)	133.8 (−1.44)	130.6 (−1.59)*^a^*	122 (−2.52)
Weight in kg (SDS)	19 (−2.86)	9.9 (−4.7)	42.3 (0.77)	27.4 (−1.32)	16.5 (−4.72)
OFC in cm (SDS)	53 (0.87)	NA	49.5 (−2.78)	53.4 (0.04)	45.8 (−5.02)
B-Relative Macrocephaly	X	X	X	—	X
Feeding difficulties	X	X	X	X	X
Protruding forehead	X	—	X	X	X
Body asymmetry	—	—	X	—	—
NH-CSS	4/6	4/6	5/6	4/6	5/6
Triangular face	X	—	—	X	X
Micrognathia	—	X	X	X	X
Thin lips	—	—	X	—	X
Down-turned mouth	—	—	—	—	—
Ogival palate	—	X	X	X	—
Other dysmorphisms	Bulbous nose, proptosis, straight eyebrows, UsPF	Anteverted nares	UsPF, strabismus, TEa	Bulbous nose, DsPF, short neck, pointed and protruding ears, indented helix	Periorbital fullness, long nose, eversion of the lower eyelid
Digital anomalies	Cli	—	Cli + Bra + Syn	Cli	Cli + Syn
Small hand/feet	X	—	X	—	X
Ligamentous laxity	—	—	X	—	—
Hypotonia	—	X	X	X	X
Psychomotor delay	—	MD + SD + ID	MD + severe SD + moderate ID	MD + SD	MD + SD + ID
Other features	Winged scapula, DBA, scoliosis, precocious puberty, two CdLS, mild bowed legs	GER, cardiomyopathy, narrow thorax, severe neutropenia, ExPa insufficiency, pancytopenia, DBA	GER, VSD, cryptorchidism, ataxic gait, DBM, cerebellar anomalies	GER, narrow larynx, choanal atresia, DBA, mixed profound/moderate deafness, severe left cochlear nerve hypoplasia	GER, elbows laxity, eosinophilic esophagitis, DBM

Abbreviations: ADHD, attention deficit hyperactivity disorder; BL, birth length; BOFC, birth occipital-frontal circumference; Bra, brachydactyly; BW, birth weight; Cli, clinodactyly of the fifth finger; DBA, delayed bone age; DBM, delayed brain myelinization; DsPF, down-slanting palpebral fissures; CdLS, cafè du lait spots; ExPa, exocrine pancreas; GER, gastroesophageal reflux; HypoE, hypoglycemic episodes; ID, intellectual disability; IUGR, intrauterine growth restriction; m, months; MD, motor delay; NA, not available; NGS, next-generation sequencing; NH-CSS, Netchine–Harbison Clinical Scoring System; OFC, occipital-frontal circumference; SD, speech delay; SDS, SD score; Syn, syndactyly of the second-third toe; TEa, tooth enamel anomalies; UsPF, up-slanting palpebral fissures; VSD, interventricular septal defect; y, year.

^*a*^Height < −2 SDS before GH treatment.

^*b*^In infancy.

Four patients, despite having a score of NH-CSS ≥ 4, revealed diagnoses due to alteration in genes associated with diseases characterized by growth retardation. SRS74 has an autosomal recessive 3-M syndrome type 3, which reassembles the clinical features of SRS, including relative macrocephaly and facial dysmorphisms, features also present in our patient. Radiological evidence of 3-M syndrome, such as broad thorax, prominent heels, and ligamentous laxity (27, 28), could not be ascertained because the child was not present at the follow-up at 4 months. Macrocephaly at birth and PNGR raised SRS suspicion for the SRS03 girl, but the *FGFR3* variant was consistent with a diagnosis of hypochondroplasia (29). In cases SRS104 and SRS84, the initial growth retardation was misleading, and the finding of a Shwachman–Diamond syndrome type 1 and of CSS12, respectively, accurately reflected the present phenotype of these children. As indicated in Table 2, patient SRS104 developed several symptoms of the multisystemic Shwachman–Diamond syndrome type 1 (30), and SRS84 showed the neurological involvement associated with CSS12 (31).

### IGF1R Analysis in SRS Patients

Given the disclosure of 2 SRS with *IGF1R* variants in our WES-enrolled patients, the gene was sequenced in the remaining 114 patients (Fig. 1). We identified 2 likely pathogenic variants inherited from affected parents and 1 maternally inherited p.(Glu1356Lys) variant, classified as VUS (Table 3) as the mother's phenotype has not been ascertained. This variant has been already reported twice (32, 33), and functional studies demonstrated a significant decrease in AKT phosphorylation in vitro (32). Additionally, intragenic deletion or duplication were ruled out for the *HMGA2* and *IGF1R* genes by MLPA analysis in all 117 negative patients and in the 2 patients with uncertain diagnosis. In the 5 *IGF1R* patients, a putative double-hit was excluded by MLPA.

**Table 3.** IGF1R variants identified in the remaining SRS patients (reference genome hg19)

Gene	Patient	Method	Coding DNA level	Protein level	Mendelian trait	Inheritance	gnomAD Exomes frequency	CADD	Polyphen	SIFT	ACMG classification
*IGF1R*	SRS02	NGS Amplicon	NM_000875: c.4066G > A	p.(Glu1356Lys)	AD, het	mat^b^	0.0000483	25.5	0.99 (D)	0.13 (T)	Uncertain significance (PS3, PM2, PP2)
*IGF1R*	SRS88	NGS Amplicon	NM_000875: c.3616G > A	p.(Ala1206Thr)	AD, het	pat^a^	0.0000159	24	0.92 (D)	0.48 (T)	Likely pathogenic (PM1, PM2, PP1, PP2, PP3, PP4)
*Igf1r*	Srs103	Ngs Amplicon	Nm_000875: C.266g > A	P.(Arg89gln)	Ad, Het	pat^a^	0.00000398	31	0.98 (D)	0 (D)	Likely Pathogenic (Pm2, Pp1, Pp2, Pp3, Pp4)

Abbreviations: ACMG, American College of Medical Genetics and Genomics; AD, autosomal dominant; D, damaging; gnomAD, Genome Aggregation Databases; het, heterozygous; mat, maternal; pat, paternal; SRS, Silver–Russell syndrome; T, tolerated.

^*a*^Affected parents

^*b*^Carrier.

### Clinical Features of the IGF1R Patients

#### Patient SRS02

This pateint was born at 31 + 4 weeks of gestation with a birth weight (BW) of 910 g (−2.3 SDS), a birth length (BL) of 33 cm (−3.29 SDS), and an occipital-frontal circumference (OFC) of 26 cm (−2.16 SDS), after a pregnancy characterized by intrauterine growth restriction (IUGR). He displayed a triangular face with a prominent forehead and frontal bossing, down-slanting of the palpebral fissures, and a bulbous nasal tip with a depressed nasal bridge and thin lips. Penoscrotal hypospadias (grade III), hydrocele, cryptorchidism, inguinoscrotal hernia, ventricular-septal defect, hypotonia, feeding difficulties, and episodes of hypoglycemia were also reported. At 5 months (3 months corrected), he showed a weight of 3.65 kg (−4.00 SDS), a length of 53 cm (−4.33 SDS), and an OFC of 39 cm (−1.82 SDS). At 11 months (9 months corrected) he showed a weight of 6.86 Kg (−2.96 SDS), a length of 66.5 cm (−2.21 SDS), and an OFC of 45.3 cm (−0.47 SDS). The heterozygous *IGF1R* variant (NM_000875):c.4066G > A p.(Glu1356Lys) was maternally inherited. Unfortunately, clinical data for the mother were not available.

#### Patient SRS67

This patient was born after a 38-week pregnancy, which was only complicated by poor fetal growth. At birth, her BW was 2100 g (−2.5 SDS), her BL was 45 cm (−2.02 SDS), and her OFC was 31 cm (−2.23 SDS). She also experienced feeding difficulties with gastroesophageal reflux, episodes of hypoglycemia, and excessive sweating. Fifth finger clinodactyly and brachydactyly were observed. Her facial features included a triangular face with a protruding forehead, micrognathia, exophthalmos with mild hypertelorism, and a thin upper lip with a downturned mouth (Fig. 2C). At 21 months of age, she weighed 7.3 kg (−5.13 SDS), measured 75 cm in height (−2.52 SDS), and had an OFC of 43 cm (−2.92 SDS). The growth chart is reported in Supplementary Fig. S2A (26). At the latest assessment at 12 years old, her weight was 26 kg (−2.46 SDS), her height was 133 cm (−2.15 SDS), and her OFC was 50.7 cm (−1.96 SDS). The patient and her mother carried the same heterozygous *IGF1R* variant (NM_000875):c.1079T > C p.(Leu360Ser). A history of perinatal and postnatal growth retardation was documented in her mother, who attained a final height of 146 cm (−2.66 SDS). Additionally, she exhibits similar facial dysmorphisms to her daughter, including the protruding forehead. Both the proband and her mother exhibited appropriate GH levels: 6.98 µg/L (range 0.12-8.05 µg/L) and 0.3 µg/L (range 0.13-9.88 µg/L), respectively. However, IGF-1 levels were elevated in the proband (912 µg/L, range 132-451 µg/L) and within a normal range in her mother (151 µg/L, range 78.7-218 µg/L).

#### Patient SRS88

IUGR was diagnosed during the pregnancy, and the patient was born at 36 + 3 weeks of gestation. Her BW was 1570 g (−2.7 SDS), her BL was 40 cm (−2.99 SDS), and her OFC was 29.5 cm (−2.39 SDS). Facial dysmorphisms included a triangular face, protruding forehead, micrognathia, thin lips, and a downturned mouth. She experienced feeding difficulties, fifth finger clinodactyly, brachydactyly, and hypotonia. At 21 months of age, she weighed 7.5 kg (−4.79 SDS), measured 71.2 cm in length (−3.61 SDS), and had an OFC of 44.2 cm (−2.04 SDS). Endocrinological evaluation showed appropriate levels of GH and IGF-1 (79 ng/mL; normal range 48-187 ng/mL). SRS88's father has the same heterozygous *IGF1R* variant (NM_000875):c.3616G > A p.(Ala1206Thr). He had a stature of 160 cm (−2.34 SDS), but, unfortunately, other clinical data were unavailable.

#### Patient SRS103

This patient was born at 37 + 4 weeks of gestation, weighing 2020g (−2.07 SDS), measuring 44 cm in length (−1.88 SDS), and with an OFC of 30 cm (−2.4 SDS), after a pregnancy characterized by IUGR. At birth, she experienced feeding difficulties with gastroesophageal reflux and fifth finger clinodactyly. Dysmorphic features included a small and triangular face with a protruding forehead and frontal bossing, thin lips, and short palpebral fissures. At 13 months of age, her weight was 6.3 kg (−4.53 SDS), her height was 69 cm (−2.13 SDS), and her OFC was 42.6 cm (−2.29 SDS). The growth chart is reported in Supplementary Fig. S2B (26). Endocrinological evaluation showed high levels of GH (14 ng/mL, range 0.14-6.27 ng/mL) and normal levels of IGF-1 (53 ng/mL, range 15-92 ng/mL). Heterozygosity for the *IGF1R* variant (NM_000875):c.266G > A p.(Arg89Gln) was found in both the patient and her father, who exhibited a similar clinical phenotype. He was born at 40 weeks of gestation with a BW of 2800 g (−1.97 SDS), a BL of 46 cm (−2.76 SDS), and an OFC of 31 cm (−3.36 SDS). At 1 year, he weighed 7.8 kg (−2.73 SDS), measured 70 cm in height (−2.23 SDS), and had an OFC of 43 cm (−2.82 SDS). His height remained stable around the third percentile from 2 years of age, reaching a final stature of 165 cm (−1.70 SDS). Facial dysmorphisms included a triangular face and protruding forehead with frontal bossing.

#### Patient SRS114

This patient was the first son of healthy parents. IUGR was diagnosed during the pregnancy. He was born at 37 weeks of gestation with a BW of 2020g (−2.48 SDS), a BL of 42 cm (−2.90 SDS), and an OFC of 31 cm (−2.07 SDS). At the age of 18 months, his weight was 7.680 kg (−4.1 SDS), his length was 74.5 cm (−2.53 SDS), and his OFC was 43.5 cm (−3.22 SDS). The growth chart is reported in Supplementary Fig. S2C (26). He displayed feeding difficulties, muscular hypotonia, fifth finger clinodactyly, and phimosis. Facial dysmorphic features included a triangular face, a protruding forehead, and micrognathia (Fig. 2D). Speech delay was observed, and a specific learning disability (dyslexia) was diagnosed later on. GH stimulation tests were inconclusive: peak GH after arginine test was pathological (1.17 ng/mL), while peak GH after glucagon test was 16.76 ng/mL (normal value >8). Basal GH was 3.14 ng/mL. IGF-1 level was normal (126 ng/mL, +0.45 SDS) at age 2 years 9 months. He started GH therapy (rhGH) at age 4 years 6 months, and his height SDS improved until normalization (last visit at 12 years 9 months: height −1.62 SDS) even though delta from target height is still slightly lower than normal (−1.77 SDS). The last head circumference was 48.8 cm (−3.51 SDS). WES analysis revealed a de novo (NM_000875):c.1363T > C p.(Cys455Arg) heterozygous variant in the *IGF1R* gene.

The clinical characteristics of our *IGF1R* patients, assessed using both the SRS and the *IGF1R* Clinical Scoring System (33), are presented in Table 4. Each patient met 4 out of 6 criteria of the NH-CSS, and 4 patients had an *IGF1R* positive score ≥3.

**Table 4.** **Clinical characteristics of the 5 IGF1R patients according to the items of both Netchine**–**Harbison Clinical Scoring System and IGF1R Clinical Scoring System**

	Netchine–Harbison Clinical Scoring System							IGF1R Clinical Scoring System				
	SGA (BW and/or BL ≤ −2 SDS)	Height at 24 months ≤ −2 SDS	Relative macrocephaly at birth	Feeding difficulties and/or BMI ≤ −2 SDS	Protruding forehead	Body asymmetry	Score	BW and/or BL < −1 SDS	Height < −2.5 SDS	HC at presentation < −2 SDS	IGF-1 SDS > 0	Score
SRS02	X	X	—	X	X	—	4/6	X	X	—	NA	2/3
SRS67	X	X	—	X	X	—	4/6	X	—	X	X	3/4
SRS88	X	X	—	X	X	—	4/6	X	X	X	X	4/4
SRS103	X	X	—	X	X	—	4/6	X	—	X	X	3/4
SRS114	X	X	—	X	X	—	4/6	X	X	X	X	4/4

Abbreviations: BL, birth length; BW, birth weight; NA, not available; OFC, occipital-frontal circumference; SGA, small for gestational age.

### (Epi)Genetic and Genetic SRS Patients at Clinical Comparison

Table 5 gives a comprehensive overview of the molecular and clinical features of *IGF2*, *PLAG1*, and *HMGA2* patients reported in the literature and this study. The last column provides molecular and clinical features of *IGF1R* patients (n = 202, including 53 symptomatic and 11 asymptomatic parents). The bibliographic sources are detailed in Supplementary Tables S1A, S1B, and 2 (26). Furthermore, we report the clinical data of our SRS cohort, IC1_LoM (n = 73) and UPD(7)mat (n = 21) in Table 5 and Supplementary Table S3 (26), respectively. The frequency of each SRS feature was evaluated in the entire group of cohorts (literature plus our data). Then we compared patients with (epi)genetic and mosaic alteration IC1_LoM vs patients with a genetic pathogenic variant in the *IGF2*-*PLAG1*-*HMGA2* axis (genetic SRS) and in the *IGF1R* gene. As shown in Table 5, *PLAG1*, *HMGA2*, and *IGF1R* patients exhibited a lower frequency of body asymmetry and of relative macrocephaly at birth and postnatal life, while *IGF2* patients displayed an increased frequency of feeding difficulties, heart defects, skeletal malformations, and developmental delay. Protruding forehead and dysmorphic facial features are less common in *IGF1R* patients. Furthermore, genetic SRS and *IGF1R* patients show postnatal microcephaly more frequently than IC1_LoM SRS.

**Table 5.** Frequency of the clinical features identified in our cohort of (epi)genetic SRS and in patients reported in the literature and in this study with *IGF2*, *PLAG1*, *HMGA2*, and *IGF1R* variants (see Supplementary Table S2)

	Our SRS cohort	*IGF2*		*HMGA2*		*PLAG1*		*IGF1R*	
	IC1_LoM (%)	Total (%)	*P*-value	Total (%)	*P*-value	Total (%)	*P*-value	Total (%)	*P*-value
Reported variants									
Truncated variant		6/19 (32)		8/19 (42)		7/9 (78)		23/108 (21)	
Splicing variant		4/19 (21)		5/19 (26)		0/9 (0)		4/108 (4)	
Missense	—	9/19 (47)		3/19 (16)		1/9 (11)		71/108 (66)	
In-frame del/ins		0/19 (0)		0/19 (0)		1/9 (11)		4/108 (4)	
Intragenic deletion		0/19 (0)		3/19 (16)		0/9 (0)		6/108 (5)	
Segregation analysis									
De novo		13/18 (72)		6/15 (40)		2/9 (22)		8/74 (11)	
Familial cases		5/18 (28)		9/15 (60)		7/9 (78)		66/74 (89)	
Symptomatic parent	—	2/5 (40)		9/9 (100)		7/7 (100)		56/68 (82)	
Asymptomatic parent		3/5 (60)		—		—		12/68 (18)	
Clinical features of evaluated patients									
SGA	56/60 (93)	23/24 (96)	ns	20/21 (95)	ns	15/15 (100)	ns	98/117 (84)	ns
PNGR	54/61 (88)	23/23 (100)	ns	21/21 (100)	ns	15/15 (100)	ns	186/202 (92)	ns
Relative macrocephaly at birth	41/52 (79)	17/22 (77)	ns	6/15 (40)	*^b^*	4/9 (44)	*^a^*	11/54 (20)	*^c^*
Feeding difficulties	39/61 (64)	23/24 (96)	*^b^*	14/17 (82)	ns	12/13 (92)	ns	55/110 (50)	ns
Protruding forehead	47/59 (79)	20/24 (83)	ns	14/20 (70)	ns	10/13 (77)	ns	21/66 (32)	*^c^*
Body asymmetry	44/61 (72)	6/24 (25)	*^c^*	1/19 (5)	*^c^*	0/14 (0)	*^c^*	1/64 (1.5)	*^c^*
SRS clinical diagnosis (NH-CCS ≥4)	61/73 (83)	20/23 (87)	ns	13/17 (76)	ns	9/10 (90)	ns	22/68 (32)	*^c^*
Intrauterine growth restriction	50/59 (84)	19/21 (90)	ns	12/13 (92)	ns	14/14 (100)	ns	44/60 (73)	ns
Dysmorphic features	50/56 (89)	21/22 (95)	ns	18/20 (90)	ns	12/13 (92)	ns	44/92 (48)	*^c^*
Microcephaly (OFC SDS < −2)	9/45 (20)	10/15 (67)	*^c^*	5/8 (62)	*^b^*	8/9 (88)	*^c^*	85/108 (79)	*^c^*
Postnatal relative macrocephaly	36/45 (80)	10/15 (67)	ns	2/8 (25)	*^b^*	1/9 (11)	*^c^*	22/81 (27)	*^c^*
Heart defects	6/48 (13)	10/22 (45)	*^b^*	0/18 (0)	ns	1/9 (11)	ns	14/110 (13)	ns
Genitalia abnormalities	7/62 (11)	7/23 (30)	ns	2/18 (11)	ns	1/9 (11)	ns	6/110 (5.5)	ns
Digital anomalies	41/57 (72)	16/22 (73)	ns	5/18 (28)	*^b^*	3/10 (30)	*^b^*	22/93 (24)	*^c^*
Skeletal malformations	2/51 (4)	5/22 (23)	*^a^*	3/18 (17)	ns	1/10 (10)	ns	9/94 (10)	ns
Motor delay	8/50 (16)	14/16 (87)	*^c^*	1/13 (8)	ns	3/9 (33)	ns	20/98 (20)	ns
Speech delay	9/50 (18)	11/16 (69)	*^c^*	1/13 (8)	ns	2/9 (22)	ns	16/89 (18)	ns
Intellectual disability	3/50 (6)	5/17 (24)	*^a^*	0/13 (0)	ns	1/9 (11)	ns	24/106 (22)	*^a^*
Endocrinological features of evaluated patients									
Delayed bone age	—	6/8 (75)		7/8 (88)		1/3 (33)		38/57 (67)	
GH levels									
Low	3/32 (9)	1/9 (11)	ns	2/5 (40)	ns	0/3 (0)	ns	3/46 (7)	ns
Normal	29/32 (91)	7/9 (78)	ns	3/5 (60)	ns	3/3 (100)	ns	36/46 (78)	ns
High	0/32 (0)	1/9 (11)	ns	0/5 (0)	ns	0/3 (0)	ns	7/46 (15)	*^a^*
Serum IGF-1 levels									
Low	—	1/16 (6)		1/13 (8)		0/6 (0)		2/102 (2)	
Normal	—	9/16 (56)		11/13 (84)		5/6 (83)		60/102 (58)	
High	—	6/16 (38)		1/13 (8)		1/6 (17)		40/102 (40)	

The frequency of the sporadic and familial cases was calculated excluding those where segregation analysis was not assessed. The familial members reported with only short stature (#) and as asymptomatic were included in the count of the PNGR in the *IGF1R* cohort. Clinical data of (epi)genetic (IC1_LoM and UPD(7)mat) and genetic SRS patients were compared using Fisher's exact test.

Abbreviations: NH-CSS, Netchine-Harbison Clinical Scoring System; ns, not significant; OFC, occipital-frontal circumference; PNGR, postnatal growth retardation; SGA, small for gestational age; SRS, Silver–Russell syndrome.

^*a*^ *P*-value ≤ .05.

^*b*^ *P*-value ≤ .01.

^*c*^ *P*-value ≤ .001.

## Discussion

The diagnosis of SRS should be based on the presence of specific features defined by the NH-CSS (1); indeed, SGA and PNGR are recurrent in several childhood syndromic disorders, making hard to pinpoint the correct suspicion. Prompted by this challenging issue, we selected a cohort of patients with NH-CSS ≥ 4 score for a multistep analysis, aiming to identify promising candidate genes.

Our molecular results highlighted the genetic heterogeneity of our cohort, as we identified pathogenic or likely pathogenic variants in known SRS genes, in genes associated with syndromes in strong differential diagnosis with SRS, as well as in genes not strictly correlated with the syndrome, reaching a diagnostic rate of 9.1%.

The role of the *IGF2*-*PLAG1*-*HMGA2* axis was confirmed revealing 1 variant in both *IGF2* (SRS type 3) and *HMGA2* (SRS type 5) genes and 2 variants in the *PLAG1* gene (SRS type 4). Summing up, according to our data, the diagnostic rate of *IGF2*-*PLAG1*-*HMGA2* variants is 3% (4/132) in undiagnosed and 1.8% (4/221) in our whole cohort of SRS with NH-CSS ≥ 4. The number of pathogenic variants reported in the SRS genes, including in this study, is still limited: 19 in the *IGF2* gene, 19 in the *HMGA2* gene, and 9 in *PLAG1*. Interestingly, our *PLAG1* patients carried 1 missense variant and 1 in-frame variant, respectively, while in the literature only 7 truncated variants have been reported (Table 5, Supplementary Table S1A and S1B) (26). Specifically, the in-frame deletion and the missense variants involve highly conserved amino acid residues, respectively, within the zinc-finger domains 6 and 7 of *PLAG1* (34). In vitro analysis revealed that these 2 domains are responsible for the recognition of the consensus binding motifs in target genes, in particular the *IGF2* P3 promoter, influencing its expression (35, 36).

A similar diagnostic rate was also detected for *IGF1R* variants, disclosing 5/132 patients (3.8%). A total of 108 *IGF1R* variants have been reported, which predominantly include missense (66%), (Supplementary Table S1A and S1B) (26). Here, we describe 4 likely pathogenic missense *IGF1R* variants never reported in the literature. Variants in the *IGF1R* gene are associated with a diagnosis of IGF-1RES (MIM#270450), an SRS differential diagnosis characterized by SGA and PNGR, proportionate microcephaly at birth and/or postnatally, and normal or high levels of serum IGF-1 (37). A highly variable phenotypic expression, even intrafamilial, is reported (32, 38-41).

The availability of a large cohort of (epi)genetic IC1_LoM and UPD(7) mat SRS and the extensive review of literature on the SRS cases with germinal variant in the axis genes (genetic SRS) allowed us to compare the phenotype associated with the (epi)genetic disorder, described in the SRS consensus, with the clinical features of patients with genetic deregulation in the same pathway (Table 5). The comparison was extended to the *IGF1R* gene. As expected, all groups showed a NH-CSS ≥ 4, sharing a significant pre- and postnatal growth retardation, even if only 32% of *IGF1R* cases reached a NH-CSS ≥ 4.

The clinical comparison highlights important evidence regarding macrocephaly and body asymmetry, considered the most pathognomonic features of the SRS phenotype. Data on relative macrocephaly at birth appear prevalent in patients with the (epi)genetic IC1_LoM (79%) and in those with *IGF2* variants (77%), while these features decrease to 40% in patients with *HMGA2* and *PLAG1* variants and fall to 20% in the *IGF1R* cohort. Similarly, postnatal relative macrocephaly is even more discrepant between (epi)genetic and genetic patients, varying from 80% of the IC1_LoM to 67% of *IGF2* cases and even lower in *HMGA2* (25%), *PLAG1* (11%), and *IGF1R* (27%) cases. Interestingly, both in genetic SRS and in *IGF1R* cases, the percentage of postnatal absolute microcephaly is significantly increased if compared to IC1_LoM (60-80% vs 18%). Table 5 shows that the frequency of body asymmetry is the most significant difference between (epi)genetic vs genetic SRS and *IGF1R* patients (73% vs 0-25%). This data underlines the association between mosaicism and body asymmetry, also described as isolated features in IC1_LoM cases (3, 15, 42). Another physical trait distinguishing the *IGF1R* cohort from the SRS patients is the facial dysmorphism described in only half of the *IGF1R* patients. Notably, the phenotype associated with *IGF2* variants appears more severe than that observed in IC1_LoM, mainly for the feeding difficulties, developmental delay, and heart anomalies.

In conclusion, our study expands the molecular landscape of SRS and underscores the importance of comprehensive molecular testing in the diagnosis of patients with suspected SRS.

In our cohort, imprinting defects account for about 33% of cases, and the figure rises to 40% in SRS patients with NH-CSS score ≥ 4. Our findings shed light on the role of SRS types of variants in the *IGF2*, *PLAG1*, and *HMGA2* genes, emphasizing their relevance in the pathogenesis of the syndrome. The study also reveals a comparable frequency of variants in the *IGF1R* gene across clinical SRS patients. Importantly, data collected in Table 5 display the high frequency of familial cases in *HMGA2* (60%), *PLAG1* (78%), and *IGF1R* (89%) patients (8, 10, 43-46), while 28% of *IGF2* variants are paternally inherited, with only 2 cases of affected fathers, including our family (47).

Overall, *IGF2*-*PLAG1*-*HMGA2* and *IGF1R* account for 3.6% of undiagnosed SRS, with NH-CSS score ≥ 4. The clinical review of the reported cases shows overlapping features between SRS and IGF-1RES patients, as well as the presence of some differences. This evidence prompted us to include *IGF1R* sequencing in the diagnostic workup for SRS. Moreover, due to the significant number of documented familial cases, with parents not necessarily displaying the phenotype, clinical parental studies and genetic counselling are recommended.

## Data Availability

The data supporting the findings of this study and the supplementary tables and figure are openly available in Zenodo at https://doi.org/10.5281/zenodo.11277975.
