# Correction to: “Distinguishing Genetic Alterations Versus (Epi)Mutations in Silver–Russell Syndrome and Focus on the *IGF1R* Gene”

**DOI:** 10.1210/clinem/dgae893

**Published:** 2025-01-08

In the above-named article by Vimercati A, Tannorella P, Guzzetti S, Calzari L, Gentilini D, Manfredini E, Gori G, Gaudino R, Antona V, Piccione M, Daolio C, Auricchio R, Sirchia F, Minelli A, Rossi E, Bellini M, Biasucci G, Raucci AR, Pozzobon G, Patti G, Napoli F, Larizza L, Maghnie M, and Russo S (*J Clin Endo Metab*. 2024; doi: 10.1210/clinem/dgae730), there was an error in the Funding section.

In the originally published article, the following sentence was excluded from the Funding section: “This work was supported by Italian Ministry of Health - Ricerca Corrente.” In the corrected article, the sentence has been added to the Funding section.

The article has been corrected online.

doi: 10.1210/clinem/dgae730

